# Extracorporeal Membrane Oxygenation—First Strategy for Acute Life-Threatening Pulmonary Embolism

**DOI:** 10.3389/fcvm.2022.875021

**Published:** 2022-06-03

**Authors:** Zhenjie Liu, Jinyi Chen, Xin Xu, Fen Lan, Minzhi He, Changming Shao, Yongshan Xu, Pan Han, Yibing Chen, Yongbin Zhu, Man Huang

**Affiliations:** ^1^Department of Vascular Surgery, The Second Affiliated Hospital of Zhejiang University School of Medicine, Hangzhou, China; ^2^Intensive Care Unit, The Second Affiliated Hospital of Zhejiang University School of Medicine, Hangzhou, China; ^3^Department of Respiratory Medicine, The Second Affiliated Hospital of Zhejiang University School of Medicine, Hangzhou, China; ^4^Medical Department, The Second Affiliated Hospital of Zhejiang University School of Medicine, Hangzhou, China

**Keywords:** pulmonary embolism, venoarterial extracorporeal membrane oxygenation, percutaneous mechanical thrombectomy, outcome, treatment

## Abstract

**Background:**

Both venoarterial extracorporeal membrane oxygenation (VA-ECMO) and percutaneous mechanical thrombectomy (PMT) are increasingly used to treat acute life-threatening pulmonary embolism (PE). However, there are little data regarding their effectiveness. This study aimed to present the short-term outcomes after managing nine patients with acute life-threatening massive or submassive PE by VA-ECMO with or without complemented PMT and propose a preliminary treatment algorithm.

**Methods:**

This study was a single-center retrospective review of a prospectively maintained registry. It included nine consecutive patients with massive or submassive pulmonary embolism who underwent VA-ECMO for initial hemodynamic stabilization, with or without PMT, from August 2018 to November 2021.

**Results:**

Mean patient age was 54.7 years. Four of nine patients (44.4%) required cardiopulmonary resuscitation before or during VA-ECMO cannulation. All cannulations (100%) were successfully performed percutaneously. Overall survival was 88.9% (8 of 9 patients). One patient died from a hemorrhagic stroke. Of the survivors, the median ECMO duration was 8 days in patients treated with ECMO alone and 4 days in those treated with EMCO and PMT. Five of nine patients (55.6%) required concomitant PMT to address persistent right heart dysfunction, with the remaining survivors (44.4%) receiving VA-ECMO and anticoagulation alone. For survivors receiving VA-ECMO plus PMT, median hospital lengths of stay were 7 and 13 days, respectively.

**Conclusions:**

An ECMO-first strategy complemented with PMT can be performed effectively and safely for acute life-threatening massive or submassive PE. VA-ECMO is feasible for initial stabilization, serving as a bridge to therapy primarily in inoperable patients with massive PE. Further evaluation in a larger cohort of patients is warranted to assess whether VA-ECMO plus PMT may offer an alternative or complementary therapy to thrombolysis or surgical thrombectomy.

**Type of Research:**

Single-center retrospective review of a prospectively maintained registry.

## Introduction

Acute massive pulmonary embolism (PE) remains one of the most common life-threatening diseases. It is characterized by sustained hypotension (systolic blood pressure <90 mm Hg for >15 min or requiring inotropic support), pulselessness, or persistent profound bradycardia (1). Although accounting for only 5% of all PEs, massive PEs are accompanied by mortality rates ranging from 25 to 60% (2). The aims of treating acute massive PE include rapid reduction of the thrombus load, immediate improvement of hemodynamic status, and reduction of the long-term risk of pulmonary hypertension (3–5).

Guidelines recommend surgical pulmonary thromboembolectomy is reserved for patients with documented central PE and refractory cardiogenic shock despite maximal supportive therapy and who have absolute contraindications to or have failed thrombolytic therapy (6). Although the outcomes have improved in the past two decades, the mortality rate of surgical embolectomy was still 19% in a recent pooled analysis (7). Clot fragmentation using a rotating pigtail catheter can result in distal embolization and worsening pulmonary hypertension (8). Optimal treatment of massive PE remains controversial.

Veno-arterial extracorporeal membrane oxygenation (VA-ECMO) is feasible for PE deemed inoperable or not amenable for interventional therapy when severe hemodynamic compromise [requiring or not requiring cardiopulmonary resuscitation (CPR)] is present or when thrombolytic therapy has failed (9). However, guidelines for ECMO use in massive PE are limited (10). There has also been a gradual acceptance of clot mitigation strategies for massive and submassive PE (5, 11). Various authors have reported the use of artery percutaneous mechanical thrombectomy (PMT) with several devices (12, 13). PMT may become first-line therapy for patients with massive PE, especially in patients with contraindications to systemic intravenous thrombolysis. More studies are required to identify patients who would benefit the most from VA-ECMO or PMT in acute massive PE and to determine the appropriate timing of cannulation and the optimal sequence of therapies.

Our institution has focused on developing a multidisciplinary rapid response team for PE, spearheaded by the Division of Vascular Surgery and the Department of Critical Care in the past 5 years. We report our institutional experience utilizing a perioperative VA-ECMO–first management algorithm in patients presenting with severe hemodynamic instability plus severe hypoxemia and/or cardiac arrest from massive PE. Thus, all patients at our institution with a clinical diagnosis of acute massive PE have been candidates for VA-ECMO cannulation as an initial adjunct intervention after starting therapeutic anticoagulation. We review our institutional experience utilizing VA-ECMO for patients presenting with severe shock and/or cardiac arrest from massive PE.

## Materials and Methods

### Study Design

**Figure 1:**
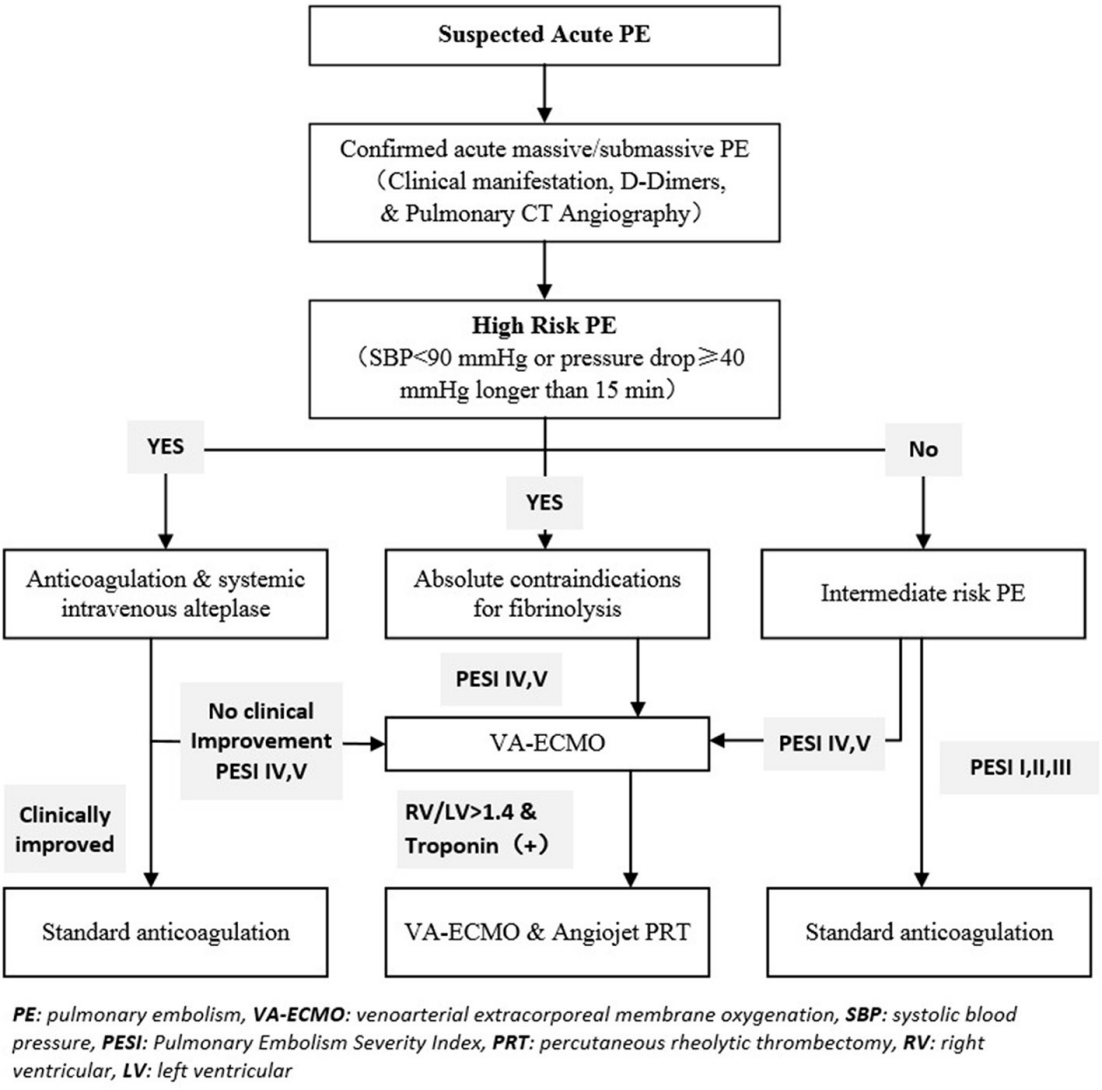
Our PE rapid response team's criteria select patients with massive or submassive PE candidates for VA-ECMO and pulmonary mechanical thrombectomy (AngioJet PRT) candidates. PE, pulmonary embolism; VA·ECMO, venoarterial extracorporeal membrane oxygenation; SBP, systolic blood pressure; PESI, Pulmonory Embolism Severity Index; PRT, percutaneous rheolytic thrombectomy; RV, right ventricular; LV, left ventricular.

This study was a single-center retrospective review of a prospectively maintained registry. We obtained institutional review board approval for this study. All patients provided informed consent according to our Research Ethics Board policy. Our algorithm for the ECMO-first strategy is summarized in Figure 1. This report includes nine consecutive patients treated at our center from August 2018 to July 2021.

During the study period, all patients at our hospital with hypotension in PE were screened for VA-ECMO suitability. The inclusion criteria for proceeding with ECMO for massive PE were as follows: (1) pre-procedure PE diagnosed by computed tomography (CT) demonstrating pulmonary clot burden and/or bedside echocardiography demonstrating evidence of pulmonary hypertension compromising right ventricular (RV) function plus the presence of hypotension and shock, or (2) an ECMO-based advanced cardiopulmonary life support program in which certain patients in cardiac arrest were placed on VA-ECMO if they were suspected of having a reversible cause (such as PE) of their arrest, the presence of which was determined by CT imaging after ECMO cannulation. Acute coronary events were common alternative causes of cardiac arrest; when present, the patients were transferred to the Cardiac Catheterization Center for coronary revascularization. VA-ECMO exclusion criteria included intracranial bleeding within the past 3 months (*n* = 1), metastatic malignancy, or age >75 years.

If there were no contraindications to fibrinolytic agents, patients with massive PE received systemic thrombolytic therapy. The strategy for systemic anticoagulation involved an intravenous heparin infusion titrated to maintain an activated partial thromboplastin time (aPTT) between 60 and 80 s.

### VA-ECMO Cannulation and Management

Patients breathing spontaneously at the PE diagnosis were preferentially cannulated using local analgesia alone to avoid the hemodynamic decline associated with mechanical ventilation. VA-ECMO was used in all nine patients in this study. Under ultrasound guidance, a 20 French (Fr)−24 Fr venous drainage cannula was placed into the common femoral vein, and a 15 Fr−18 Fr arterial cannula was inserted into the common femoral artery. After cannulation, a focused neuromotor examination of the lower extremities was performed in awake patients to identify those requiring reperfusion cannulas. Intubated patients were monitored by serial physical examinations and near-infrared spectroscopy placed on the lower extremities to detect asymmetric limb perfusion. When patients were placed on VA-ECMO, interval echocardiography was performed, and myocardial enzymes were measured to assess RV function and compromised myocardial blood supply.

### Percutaneous Mechanical Thrombectomy Procedure

**Figure 2:**
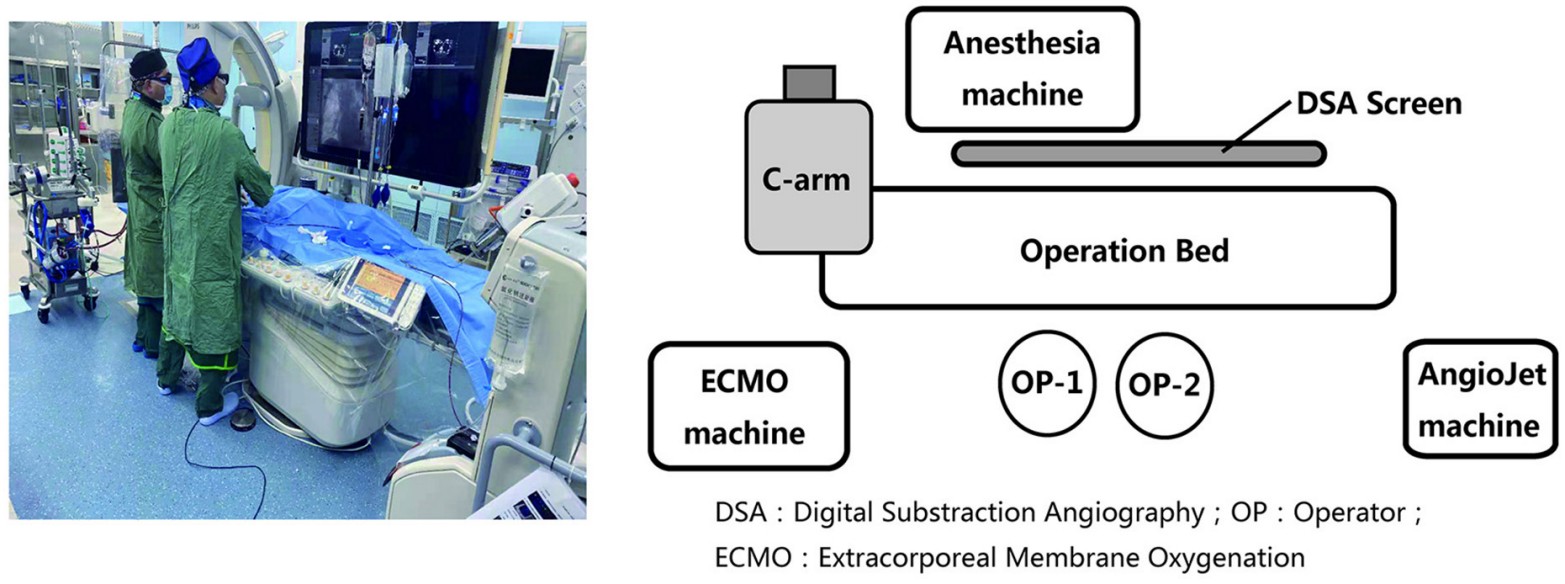
Percutaneous mechanical thrombectomy procedure with patients receiving general anesthesia continued VA-ECMO circulation in the hybrid operating room. DSA, Digital Substraction Angiography; OP, Operator; ECMO, Extracorporeal Membrane Oxygenation.

Patients presenting with an elevated troponin and an RV/left ventricular (LV) diameter ratio >1.4 were candidates for PMT *via* the Solent Omni AngioJet device (Boston Scientific, Marlborough, MA, USA) to decrease the thrombus burden. We performed PMT in a hybrid operating room with the patients receiving general anesthesia and continued VA-ECMO circulatory support (Figure 2). We planned the procedure approach by first reviewing CT scans to identify vessels with more thrombus burden so that treatment could be focused on these areas. Patients who underwent PMT were continued on heparin during the procedure.

The technique for pulmonary PMT using AngioJet has been described previously (5, 14). Patients were transported from the intensive care unit (ICU) to our hybrid operating room. After site preparation, the right common femoral vein was accessed with a 6 Fr sheath. A 5 Fr angled pigtail catheter was advanced to the main pulmonary artery and then exchanged over a standard J-wire for a 6 Fr 90-cm guiding sheath (Cook Group, Bloomington, IN, USA). To confirm the proximal occlusion site, selective angiography was performed *via* the guiding catheter or by reintroducing a pigtail catheter. Next, a guidewire (Terumo Medical, Somerset, NJ, USA) was placed beyond the thrombus with a multipurpose catheter. The AngioJet device was then advanced over the guidewire and across the occlusion in the inactivated state. Angiography was performed *via* the device to visualize the size of the vessel distal to the occlusion. If the vessel was at least 3 mm in diameter, the device was pulled back proximal to the clot and re-advanced through the clot after being activated. If the vessel was <3 mm, the wire was withdrawn and repositioned, or more commonly, the device was activated only until the vessel diameter was estimated to be at least 3 mm. Routinely, only one or two passes were made, and angiography was repeated to ensure clot extraction. The goal of therapy was never to attempt complete clot extraction or achieve an angiographically desirable result but to decrease the clot burden to improve oxygenation and hemodynamics. Pulmonary arterial pressures were measured during the procedure.

Patients were administered 70 units/kg of intravenous unfractionated heparin on arrival in the hybrid operating room, followed by additional doses titrated to maintain an activated clotting time >200 s. Patients with active bleeding or trauma with shock were administered PMT without heparin. Inferior vena cava (IVC) filters were implanted individually at the end of the PMT procedure, and the possibility of filter removal was discussed at the first follow-up visit after hospital discharge.

Hemodynamics and oxygenation were monitored continuously during the PMT procedure, and patients were transferred to the ICU for continuous monitoring after the completion of the procedure. Patients received therapeutic unfractionated heparin for 3–5 days, titrated to maintain an aPTT of 60–100 s. Patients were monitored for improvements in end-organ function and hemodynamics. Oral anticoagulation therapy was continued for at least 1 year.

### VA-ECMO Weaning

After PMT and 1–2 days of VA-ECMO support, an attempt was made to reduce VA-ECMO flow rates. Hemodynamic parameters were monitored to assess tolerance of flow reduction. Patients who tolerated minimal VA-ECMO flows and had reasonable residual RV function were decannulated from VA-ECMO *via* the percutaneous vascular access closure technique using Perclose ProGlide (Abbott Vascular, Santa Clara, CA, USA) (15).

### Study Endpoints

Clinical success was defined as the absence of death and the relief of acute symptoms. Technical success was defined as decreasing the clot burden to improve oxygenation and hemodynamics. Major complications were defined as PE-related death, and ECMO-related complications were defined as acute kidney injury (AKI), AKI requiring renal replacement therapy (RRT), stroke, bleeding, or vascular injury. PMT-related complications were hemolysis, suspected perforation, bradycardia, asystole, or thrombocytopenia. Acute kidney injury was defined as an absolute increase in serum creatinine of more than or equal to 0.3 mg/dl (≥ 26.4 μmol/l), a percentage increase in serum creatinine of more than or equal to 50% (1.5-fold from baseline), or a reduction in urine output (documented oliguria of <0.5 ml/kg per hour for more than 6 h) (16, 17).

### Follow-Up and Statistical Analysis

Repeat echocardiography was performed 3–6 months post-PMT to assess the RV/LV diameter ratio and pulmonary arterial pressures. Clinical follow-up data for at least 3 months were available for all patients. Statistical analysis was conducted using the Mann–Whitney *U*-test. *P*-values <0.05 were considered statistically significant.

## Results

### Patient Characteristics

From August 2018 to July 2021, 32 patients with high-risk acute PE were admitted to our ICU. Of these, nine were treated with VA-ECMO according to our institution's PE rapid response team's criteria for selecting massive or submassive PE candidates for VA-ECMO. Baseline characteristics of the nine included patients are summarized in Table 1. The mean patient age was 54.7 years, and seven patients (77.8%) were male. All nine patients presented with dyspnea, chest pain, hypoxia, and RV dysfunction. Eight patients (88.9%) had presyncope or syncope and hypoxia. Five patients presented with elevated troponin I and abnormal echocardiography. CT angiography revealed a central branch embolus in all patients, which was massive in five patients and submassive in four patients. Seven patients had contraindications to thrombolysis, such as recent major surgery, recent trauma with shock, malignant tumor, active bleeding, or recent stroke (Table 1).

**Table 1:** Baseline demographics, clinical characteristics, and thrombolysis contraindications of all treated patients.

**Variable**	**No. (%)**
Age, years, mean (range)	54.7 (27–72)
Sex
Male	7 (77.8)
Female	2 (22.2)
Symptoms
Dyspnea	9 (100)
Chest pain	9 (100)
Presyncope or syncope	8 (88.9)
Clinical findings at presentation
Hypoxia	9 (100)
Hypotension	8 (88.9)
Right ventricular dysfunction	9 (100)
Troponin I > 0.01 ng/mL	5 (55.6)
Echocardiography
Right ventricular dilation^a^	5 (55.6)
Abnormal interventricular septal motion	5 (55.6)
Computed tomography angiography
Main branch embolus	9 (100.0)
Massive pulmonary embolus	5 (55.6)
Submassive pulmonary embolus	4 (44.4)
Contraindications to thrombolysis
Recent major surgery	4 (44.4)
Recent trauma with shock	3 (33.3)
Malignant tumor	1 (11.1)
Active bleeding	1 (11.1)
Recent stroke (<14 days)	1 (11.1)
Comorbidities
Hypertension	2 (22.2)
Diabetes	1 (11.1)
Coronary artery disease	1 (11.1)

a *Right ventricular/left ventricular diameter ratio >1.40*.

Four of the nine patients (44.4%) included in this study had a cardiac arrest before VA-ECMO cannulation (Table 2). Seven patients (77.8%) had an identifiable inciting cause of their PE, such as recent surgery (*n* = 4) or recent trauma (*n* = 3) (Table 3). Two patients with acute massive PE had idiopathic hypercoagulability and received VA-ECMO because of failure to improve clinically after anticoagulation and systemic intravenous alteplase. Of these two patients, one required transfusion secondary to a large-volume gastric hemorrhage, which was confirmed by gastric endoscopy to originate from a gastric ulcer, and the other experienced no complications while receiving concurrent VA-ECMO and PMT.

**Table 2:** Clinical and laboratory characteristics of patients before VA-ECMO cannulation.

**Variable**	**Value (*N* = 9)**
Cardiac arrest	4 (44.4)
Hemodynamics at the time of cannulation
Heart rate, beats/min	125 (112–134)
Systolic blood pressure, mm Hg	79.89 ± 20.94
Cardiac blood tests
NT-proBNP, pg/mL	6,535 ± 11,098
Troponin I, ng/mL	0.96 ± 1.13
Respiratory status at the time of cannulation
Respiratory rate, breaths/min	23 (19–29)
FiO_2_, %	78.9 ± 26.2
Intubation	9 (100)
Arterial blood gases
PaO_2_, mm Hg	78.2 (57.8–119.6)
pH	7.18 (7.02–7.27)
PESI score	130 (110–140)

*Values are n (%), median (range), or mean ± standard deviation*.

*NT-proBNP, N-terminal pro B-type natriuretic peptide; FiO_2_, inspired oxygen fraction; PESI, Pulmonary Embolism Severity Index*.

**Table 3:** Characteristics of each patient treated with VA-ECMO with or without AngioJet percutaneous rheolytic thrombectomy.

**Sex/age (years)**	**Massive/submassive**	**Etiology**	**Clinical manifestations**	**RV/LV diameter ratio**	**PESI score**	**Thrombolysis contraindicated**	**Treatment**
F/60	Submassive	Recent surgery, recent stroke	Dyspnea, chest pain, syncope, cardiac arrest	1.01	110	No	ECMO
M/57	Submassive	Lung cancer, recent surgery	Dyspnea, chest pain, presyncope	1.12	110	No	ECMO
F/27	Massive	Recent trauma with shock	Dyspnea, chest pain, presyncope	1.20	110	Yes	ECMO
M/40	Massive	Recent trauma with shock	Dyspnea, chest pain, syncope, shock	1.13	120	Yes	ECMO
M/32	Massive	Idiopathic hypercoagulability	Dyspnea, chest pain, syncope, cardiac arrest	1.67	140	No	ECMO+PMT
M/67	Submassive	Recent surgery, active bleeding	Dyspnea, chest pain, presyncope	1.53	130	Yes	ECMO+PMT
M/69	Massive	Recent surgery	Dyspnea, chest pain, cardiac arrest	1.47	130	No	ECMO+PMT
M/72	Massive	Idiopathic hypercoagulability	Dyspnea, chest pain, presyncope	1.43	140	No	ECMO+PMT
M/68	Submassive	Recent trauma with shock	Dyspnea, chest pain, cardiac arrest	1.40	130	Yes	ECMO+PMT

*VA-ECMO, venoarterial extracorporeal membrane oxygenation; PMT, percutaneous mechanical thrombectomy (AngioJet percutaneous rheolytic thrombectomy); RV, right ventricular; LV, left ventricular; PESI, Pulmonary Embolism Severity Index; F, female; M, male*.

Table 2 summarizes the clinical and laboratory characteristics of the patients at the time of VA-ECMO cannulation. All patients were intubated and either hypotensive or required vasopressors at the time of cannulation. VA-ECMO cannulation was technically successful in all nine patients. Four of the nine patients (44.4%) required CPR before or during ECMO cannulation. Two cardiac arrests occurred while the patients before being admitted to the hospital; one of these two patients survived. The cohort of patients presented with a mean (± standard deviation) N-terminal pro-B-type natriuretic peptide of 6,535 ± 11,098 pg/mL, median pH of 7.18 (range, 7.02–7.27), and mean troponin I of 0.96 ± 1.13 ng/mL. The PE Severity Index scores ranged from 110 to 140, with a median of 130.

### Effects of Initiating VA-ECMO

Heart rate, systolic blood pressure, and oxygenation improved significantly immediately after initiating VA-ECMO. One patient required transfusion for underlying traumatic injuries in the first 24 h after VA-ECMO cannulation. Two patients developed AKI and required continuous RRT. Two patients (22.2%) required reperfusion cannulas for limb ischemia. Limb loss did not occur in any of the nine patients. The mean time to initiate ECMO from the onset was not recorded in our patient registry. Seven patients (77.8%) required anticoagulation alone for definitive PE management, while two patients without contraindications to thrombolytics also received systemic intravenous alteplase. Table 3 summarizes the characteristics of each patient treated with VA-ECMO with or without AngioJet percutaneous rheolytic thrombectomy.

### Effects of Percutaneous Mechanical Thrombectomy

**Figure 3:**
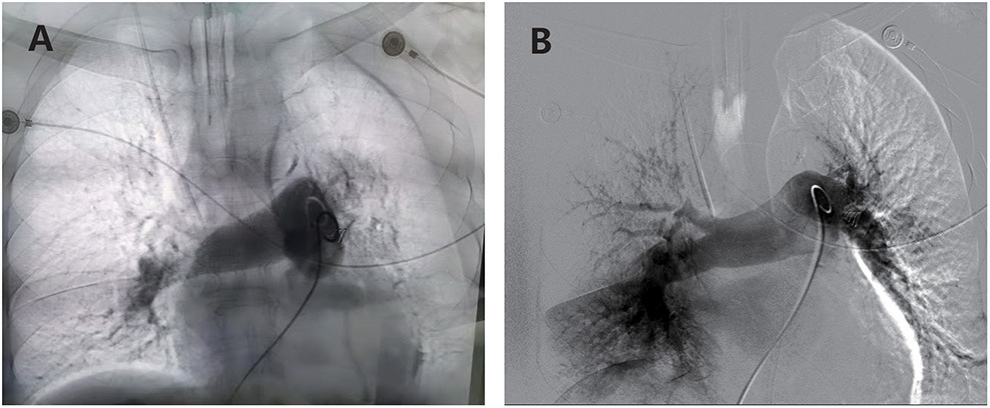
**(A)** Angiography demonstrating extensive thrombus in bilateral pulmonary arteries with compromised blood flow. **(B)** Angiography post-thrombectomy demonstrates decreased clot burden and improved flow in both pulmonary arteries.

Five of the nine patients (55.6%) underwent PMT because of profound hemodynamic instability, with a bedside echocardiography RV/LV diameter ratio >1.40 and elevated troponin. At PMT, four patients required support with vasopressors, and all five patients required mechanical ventilation. In all attempted cases, the rheolytic thrombectomy catheter was successfully inserted and operated on *via* a 0.035-inch guidewire. Table 4 summarizes the hemodynamic parameters of all five patients before and after ECMO plus PMT. Hemodynamic status improved in all patients, as evidenced by significant improvements in all of the following: median invasive systolic pulmonary arterial pressure (65 vs. 45 mm Hg, *P* < 0.05), median pH (7.32 vs. 7.38; *P* < 0.005), median mid-RV diameter (58 vs. 52 mm; *P* < 0.001), and median RV/LV diameter ratio (1.47 vs. 1.08). Figure 3 shows representative angiographic images before and after PMT.

**Table 4:** Hemodynamic parameters before and after percutaneous mechanical thrombectomy (*n* = 5).

**Parameter**	**Before PMT**	**After PMT**	***P*-value**
Clinical status
Heart rate, bpm	105 (103–120)	99 (91–127)	0.586
Heart rate >100 bpm	5/5 (60)	2/5 (40)	
Systolic blood pressure (mm Hg)	91 (75–127)	95 (81–120)	0.403
Systolic blood pressure	2/5 (40)	1/5 (20)	
< 90 mm Hg
Invasive systolic pulmonary	65 (38–70)	45 (35–55)	**0.040**
arterial pressure (mm Hg)
Laboratory results
pH	7.32 (7.18–7.42)	7.38 (7.25–7.42)	**0.040**
Oxygen saturation, %	98 (95–100)	99 (96–100)	0.145
Doppler echocardiography
Mid-RV diameter, mm	58 (56–67)	52 (50–62)	**0.001**
RV/LV diameter ratio	1.47 (1.40–1.67)	1.08 (1.03–1.47)	**0.001**
FiO_2_, %	80 (60–100)	80 (60–100)	0.178
Number of vasopressors	1 (0–2)	1 (0–2)	0.374

*Data are presented as median (range) or number (percentage)*.

*PMT, percutaneous mechanical thrombectomy; bpm, beats per minute; RV, right ventricular; LV, left ventricular; FiO_2_, inspired oxygen fraction*.

*Invasive pulmonary arterial pressure was measured with a pulmonary arterial catheter. Bold indicates p value < 0.05*.

No death attributed to the PMT procedure occurred in any five patients. All patients developed transient bradycardia during AngioJet device activation. Four patients also developed hemolysis during PMT. No patient required atropine or pacing, and the bradycardia self-terminate upon cessation of device activation. The median procedure duration was 58 min. After thrombectomy, two patients with idiopathic hypercoagulability received adjuvant reteplase thrombolysis to treat residual thrombus. An IVC filter was inserted because of an iliac venous floating thrombus in one patient who underwent VA-ECMO plus PMT and in one patient who underwent VA-ECMO alone. After the PMT procedure, RV size improved in four patients (44.4%). In one patient, the RV size was moderately increased, compared to only mild dilation prior to PMT. On the whole, RV function improved in 6 of 9 patients (66.7%) and remained unchanged in three patients after treatment.

### Survival, Success, and Complications

The overall survival rate was 88.9% (8 of 9 patients), with one death resulting from hemorrhagic stroke. Technical success was achieved in all nine patients (100%). Table 5 summarizes clinical outcomes and complications of patients with acute massive or submassive PE receiving VA-ECMO with or without PMT. One patient with a hemorrhagic stroke died after the family decided to withdraw care because of poor neurologic status and multiorgan failure. In the survivors, the median VA-ECMO duration was 8 days in patients treated with VA-ECMO alone and 4 days in those treated with VA-ECMO plus PMT. The median time from ECMO cannulation to discontinuation of all vasopressors was 12 h. There was no apparent association between ECMO duration and ECMO-related complications. No vascular injury was noted, as all patients underwent Perclose ProGlide closure for decannulation.

**Table 5:** Clinical outcomes and complications of patients with acute massive or submassive pulmonary embolism treated with VA-ECMO with or without AngioJet rheolytic thrombectomy.

**Outcomes and complications**	**VA-ECMO only (*n* = 4)**	**VA-ECMO plus AngioJet (*n* = 5)**
Clinical success	4	4
Technical success	4	5
ECMO duration, days	8 (2–14)	4 (2–11)
PMT procedure duration, minutes	–	58 (45–83)
PE-related death	0	1
IVC filter placement	1	1
In-hospital and 90-day survival	4	4
Hospital length of stay, days	15 (5–24)	13 (8–17)
ECMO and ICU complications (*n* = 9)
AKI	0	2
AKI requiring RRT	0	1
Stroke	0	1
Hemorrhage requiring transfusion	0	1
Vascular injury	0	0
Percutaneous mechanical thrombectomy (*n* = 5)
Hemolysis	–	5
Suspected perforation	–	0
Bradycardia	–	5
Asystole	–	2
Thrombocytopenia	–	0

*Values are numbers or median (range)*.

*ECMO, extracorporeal membrane oxygenation; PMT, percutaneous mechanical thrombectomy; PE, pulmonary embolism; IVC, inferior vena cava; ICU, intensive care unit; AKI, acute kidney injury; RRT, renal replacement therapy; CT, computed tomography*.

The eight patients who survived had continued improvement in PE symptoms. The median hospital length of stay was 15 days (range, 5–24 days) in patients treated with VA-ECMO only and 13 days (range, 8–17 days) in those treated with VA-ECMO plus PMT. Two of the nine patients (22.2%) underwent tracheostomy for prolonged ventilator dependence. Two patients were transferred to a rehabilitation or long-term care facility at discharge, and six were discharged home. Echocardiography on the day of discharge showed that pulmonary arterial systolic pressures had decreased to 38.5 ± 12.4 mm Hg in seven patients. The other survivor had no detectable tricuspid regurgitation, so pulmonary arterial pressures could not be calculated. RV size and function were normal in four of the eight survivors.

There was no additional death when assessed at 6 months or the longest follow-up time after discharge. The eight survivors underwent surveillance echocardiography 3 months after hospital discharge. RV function was normal in seven patients (77.8% of the nine original patients) and moderately reduced in one patient (11.1% of the nine patients). No survivor required home oxygen at the time of discharge.

## Discussion

### Results in Context

Massive PE remains associated with significant morbidity and mortality despite advances in treatment (18). PE presents with shock or hypotension in 5–10% of patients. Signs of RV dysfunction and injury portend a poorer prognosis (19, 20). The most important finding of this study is that an ECMO-first strategy effectively salvaged unstable patients with acute massive or submassive PE. Moreover, this study confirms the feasibility, efficacy, and relative safety of VA-ECMO complemented with AngioJet rheolytic thrombectomy for treating acute massive and submassive PE.

VA-ECMO is the primary support configuration for severe right heart failure secondary to PE, providing immediate RV decompression and augmenting cardiac output in patients with organ dysfunction. However, guidelines for ECMO use in massive PE are limited (10). ECMO was used as a bridge therapy between the primary hospital and the tertiary hospital for a more intensive treatment (21). The surgical treatment for acute massive PE has had improved outcomes in experienced centers where a rapid response multidisciplinary team has developed protocols to treat the PE patients with massive pulmonary embolism and selectively used ECMO to restore oxygenation and circulation in patients with hemodynamic collapse (22, 23). Outcomes of surgical thromboembolectomy have improved in the past decades, with mortality declining from 30% to well below 10% in centers with appropriate expertise (20, 24–26). Large randomized controlled trials, although desirable, would be challenging to conduct. Despite a high proportion (44.4%) of our patient population requiring CPR at presentation, they had a very good overall survival rate of 88.9%. These results of high-risk PE patients treated with an ECMO-first strategy are better than those of the recently published case series by Corsi et al. (27). Those authors reported a 47% 90-day survival rate in 17 patients requiring VA-ECMO for massive PE (27).

At our institution, ECMO is available at all times (24 h per day, 7 days per week) for cardiovascular and/or respiratory assistance. In our experience, ECMO can rapidly restore systemic perfusion and oxygenation. VA-ECMO support likewise mitigates the consequences of potentially fatal arrhythmias that are common within the first 24 h of hospitalization. The current study is a single-center case series that reported demographics and outcomes of patients with massive or submassive PE who underwent VA-ECMO and AngioJet rheolytic thrombectomy. Other noteworthy findings were that patients with massive PE who received VA-ECMO and PMT had satisfying outcomes from this combined management approach and that some survivors required ECMO and PMT combined therapy. Thus, based on the increased number of case reports and current practice in many centers, guidelines should evolve to consider ECMO as first-line therapy in patients with massive PE in whom classical thrombolytic therapies are restricted (1, 10, 28). The significant success we achieved following the initiation of VA-ECMO in our patients was related to the implementation of this policy at our institution.

We did perform a PMT-only strategy to rescue the patients with acute massive PE and anticoagulation contradiction in our center before starting the VA-ECMO application. From the timeliness of treatment, we prefer VA-ECMO to PMT for patients with acute massive PE and anticoagulation contradiction after 3 years of practice. The door-to-bed time of PMT usually exceeds 2 h before the epidemic in our medical center. In the meantime, we can perform VA-ECMO for patients with acute massive PE in the emergency room in less than half an hour. VA-ECMO also provides a bridge to recovery or lifesaving intervention, such as rheolytic thrombectomy.

Several PMT devices are currently available, but massive PE is an off-label indication for all of them. Most devices have been studied only in small retrospective series and highly selected patient groups (4, 5, 29, 30). This study evaluated PMT results using a rheolytic thrombectomy catheter in the pulmonary arteries. We used the AngioJet Solent Omni catheter in all patients, which is well-suited to perform PMT in this anatomical location. It allows passage *via* a 0.035-inch guidewire and provides effective clot retrieval. It is particularly efficacious for treating mainstem or saddle pulmonary emboli, for which a less potent device may not achieve adequate clot retrieval because of the larger vessel diameter or extensive thrombus burden. A more effective mechanical device can also obviate the need for thrombolytic therapy (5). However, the device must be used with caution, as activation in small distal vessels may lead to vessel trauma and rupture (5).

The AngioJet device performed well in our high-risk patient population, with a low complication rate. Several prior reports using the device for high-risk patients with PE noted various complications, including bradyarrhythmias, cardiac arrest, hemoglobinuria, temporary renal insufficiency, hemoptysis, and procedure-related deaths (4, 29, 31–34). Additional concerns related to hemolysis caused by PMT are the development of severe hyperkalemia and hemoglobinuria. We achieved marked improvements in hemodynamic and respiratory status and normalization of heart rate, blood pressure, and oxygenation with PMT in our patients. All patients were weaned off vasopressors and respiratory support after the PMT procedure. Overall, the procedure was well-tolerated with limited minor complications and no major complications.

All five patients developed hemolysis and hemoglobinuria during or after AngioJet rheolytic thrombectomy. Four of these patients developed some form of bleeding complication, including hemoptysis, hemoglobinuria, or suspected perforation. Hyperkalemia may exacerbate electrical instability, and hemoglobinuria may cause further deterioration of kidney function in these patients, who are already at risk of kidney failure because of hemodynamic instability and contrast medium administered for the PMT procedure, with or without diagnostic CT angiography (35). Placement of a temporary pacemaker wire and aggressive control of potassium levels is unnecessary in patients presenting with massive PE during the PMT procedure with the VA-EMCO support.

Reported bleeding rates of patients with massive PE treated with thrombolytics have been as high as 24% (2). The risk of major bleeding is almost double in patients with massive PE treated with fibrinolysis (36). In our series, four of the five patients who underwent PMT developed hemoptysis that resolved spontaneously, which was likely the result of brisk distal reperfusion after successful PMT of a large occlusive thrombus. Perforation of the pulmonary vessels was unlikely, as no dissection or extravasation of contrast was noted by angiography or chest CT performed within 1 h after the procedure. Potential causes of hemoptysis associated with PMT include vessel wall disruption caused by the thrombectomy device, hemorrhagic conversion of a pulmonary infarct, reperfusion injury, or formation of pulmonary arteriovenous fistula (5, 32, 37).

All of our patients experienced PMT-related bradyarrhythmias, which were self-limited and required no specific treatment. One patient required transfusion for large-volume gastric bleeding from a gastric ulcer, likely a stress-induced ulcer. Unlike prior reports, we observed no decline in renal function. The lack of procedure-related severe complications was likely partially attributed to the operator being careful to avoid aggressive device manipulation and activation times. The goal of PMT was always clinical and hemodynamic improvement rather than complete clot extraction on angiography. Repeated passes and prolonged run times are associated with more bradyarrhythmias, hemoglobinuria, and thrombocytopenia.

One patient with acute massive PE had angiographic improvement in thrombus burden with PMT but later died after the family decided to withdraw care because of poor neurologic status resulting from hemorrhagic apoplexy. Persistently elevated pulmonary arterial pressures were likely due to chronic thromboembolic pulmonary hypertension, probably due to previous PEs. One patient with acute and chronic PE had poor thrombus extraction with the AngioJet device. The device probably does not perform well in chronically organized clots without adjunctive thrombolytic therapy. This patient had persistent pulmonary hypertension and RV dilation.

### Limitations

Our study has several limitations. The data were collected retrospectively, leading to all the inherent shortcomings of a retrospective study. Furthermore, a small sample of a heterogeneous group of patients may factor in influencing results. In addition, there were no data for patients who did not receive VA-ECMO or patients who received PMT only, which would be an essential comparator group. Therefore, the conclusions drawn from this study need to be interpreted with caution. Seven patients had contraindications for thrombolytic therapy. Although VA-ECMO and PMT may be more efficacious when combined with thrombolytic therapy, the AngioJet device appears to perform well in the setting of acute thrombus without thrombolysis. Another limitation was that one operator performed all procedures, raising concerns about reproducibility. However, the techniques used are easily performed by an operator familiar with the device and angiographic anatomy of the pulmonary circulation.

Additionally, repeat angiography or CT was not routinely performed to assess the degree of thrombus burden following PMT. Consequently, we have no objective information about the degree of reduction in thrombus burden achieved by PMT. Finally, our data do not provide firm information about the superiority of the ECMO-first (with or without PMT) strategy compared to other treatments for massive PE, such as thrombolytic therapy or open surgical embolectomy.

## Conclusion

Acute massive PE carries a substantial mortality burden, and VA-ECMO provides a bridge to recovery or lifesaving intervention with satisfying outcomes. A VA-ECMO–first policy effectively salvaged unstable patients with massive PE. PMT with VA-ECMO support using the AngioJet system appears to be a promising treatment option for patients with massive proximal PE who are hemodynamically unstable and may not be eligible for thrombolytic therapy or surgical embolectomy. We propose a preliminary treatment algorithm for acute life-threatening PE with an ECMO-first strategy (Figure 1). We recommend that a multidisciplinary team evaluate patients with massive PE, and experienced operators perform PMT quickly in a facility capable of dealing with signficant medical and surgical emergencies.

## Data Availability Statement

The raw data supporting the conclusions of this article will be made available by the authors, without undue reservation.

## Ethics Statement

The studies involving human participants were reviewed and approved by the Ethics Committee of the second affiliated hospital, Zhejiang University. The patients/participants provided their written informed consent to participate in this study.

## Author Contributions

ZL, YZ, and MHu: study conception, design, and final approval of the article, and overall responsibility. ZL, JC, XX, FL, MHe, CS, YX, PH, YC, YZ, and MHu: analysis, interpretation, and approved the submitted version. ZL, XX, FL, MHe, CS, YX, PH, YC, and JC: data collection. ZL, JC, XX, YZ, and MHu: critical revision of the article. ZL and MHu: obtained funding. All authors contributed to the article and approved the submitted version.

## Funding

This work was supported by the National Natural Science Foundation of China (81670433 and 81970398), the Medical Science and Technology Project of Zhejiang Province (2020RC014), and the Natural Science Foundation of Zhejiang Province (Q20H020059), Science Fund for Distinguished Young Scholars of Zhejiang Province (LR22H020002). This work was also supported by all dedicated intensivists, vascular surgeons, perfusionists, and nurses at our institution not listed as authors.

## Conflict of Interest

The authors declare that the research was conducted in the absence of any commercial or financial relationships that could be construed as a potential conflict of interest.

## Publisher's Note

All claims expressed in this article are solely those of the authors and do not necessarily represent those of their affiliated organizations, or those of the publisher, the editors and the reviewers. Any product that may be evaluated in this article, or claim that may be made by its manufacturer, is not guaranteed or endorsed by the publisher.

## References

[1] Guliani S Das Gupta J Osofsky R Kraai EP Mitchell JA Dettmer TS. Venoarterial extracorporeal membrane oxygenation is an effective management strategy for massive pulmonary embolism patients. J Vasc Surg Venous Lymphat Disord. (2021) 9:307–14. 10.1016/j.jvsv.2020.04.033 32505687

[2] Kucher N Rossi E De Rosa M Goldhaber SZ. Massive pulmonary embolism. Circulation. (2006) 113:577–82. 10.1161/CIRCULATIONAHA.105.592592 16432055

[3] Ferrigno L Bloch R Threlkeld J Demlow T Kansal R Karmy-Jones R. Management of pulmonary embolism with rheolytic thrombectomy. Can Respir J. (2011) 18:e52–8. 10.1155/2011/614953 22059183 PMC3205106

[4] Li K Cui M Zhang K Liang K Liu H Zhai S. Treatment of acute pulmonary embolism using rheolytic thrombectomy. Eurointervention. (2021) 17:e158–66. 10.4244/EIJ-D-20-00259 32863245 PMC9725013

[5] Das S Das N Serota H Vissa S. A retrospective review of patients with massive and submassive pulmonary embolism treated with AngioJet rheolytic thrombectomy with decreased complications due to changes in thrombolytic use and procedural modifications. Vascular. (2018) 26:163–8. 10.1177/1708538117722728 28828935

[6] McMurray JJ Adamopoulos S Anker SD Auricchio A Bohm M Dickstein K. ESC guidelines for the diagnosis and treatment of acute and chronic heart failure 2012: the task force for the Diagnosis and Treatment of Acute and Chronic Heart Failure 2012 of the European Society of Cardiology. Developed in collaboration with the Heart Failure Association (HFA) of the ESC. Eur J Heart Fail. (2012) 14:803–69. 10.1093/eurjhf/hfs105 22828712

[7] Samoukovic G Malas T deVarennes B. The role of pulmonary embolectomy in the treatment of acute pulmonary embolism: a literature review from 1968 to 2008. Interact Cardiovasc Thorac Surg. (2010) 11:265–70. 10.1510/icvts.2009.228361 20547704

[8] Nakazawa K Tajima H Murata S Kumita SI Yamamoto T Tanaka K. Catheter fragmentation of acute massive pulmonary thromboembolism: distal embolisation and pulmonary arterial pressure elevation. Br J Radiol. (2008) 81:848–54. 10.1259/bjr/93840362 18941044

[9] Kmiec L Philipp A Floerchinger B Lubnow M Unterbuchner C Creutzenberg M. Extracorporeal membrane oxygenation for massive pulmonary embolism as bridge to therapy. ASAIO J. (2020) 66:146–52. 10.1097/MAT.0000000000000953 30720492

[10] Hockstein MA Creel-Bulos C Appelstein J Jabaley CS Stentz MJ. Institutional experience with venoarterial extracorporeal membrane oxygenation for massive pulmonary embolism: a retrospective case series. J Cardiothorac Vasc Anesth. (2021) 35:2681–5. 10.1053/j.jvca.2020.12.045 33531193

[11] Jaff MR McMurtry MS Archer SL Cushman M Goldenberg N Goldhaber SZ. Management of massive and submassive pulmonary embolism, iliofemoral deep vein thrombosis, and chronic thromboembolic pulmonary hypertension: a scientific statement from the American Heart Association. Circulation. (2011) 123:1788–830. 10.1161/CIR.0b013e318214914f 21422387

[12] Uflacker R. Interventional therapy for pulmonary embolism. J Vasc Interv Radiol. (2001) 12:147–64. 10.1016/S1051-0443(07)61821-1 11265879

[13] Fava M Loyola S Huete I. Massive pulmonary embolism: treatment with the hydrolyser thrombectomy catheter. J Vasc Interv Radiol. (2000) 11:1159–64. 10.1016/S1051-0443(07)61357-8 11041472

[14] Engelberger RP Kucher N. Catheter-based reperfusion treatment of pulmonary embolism. Circulation. (2011) 124:2139–44. 10.1161/CIRCULATIONAHA.111.023689 22064957

[15] Liu Z Xu Y Xu X He M Han P Shao C. Comparison of success rate and complications of totally percutaneous decannulation in patients with veno-arterial extracorporeal membrane oxygenation and endovascular aneurysm repair. Front Med. (2021) 8:724427. 10.3389/fmed.2021.724427 34490310 PMC8417572

[16] Ronco C Ricci Z De Backer D Kellum JA Taccone FS Joannidis M. Renal replacement therapy in acute kidney injury: controversy and consensus. Crit Care. (2015) 19:146. 10.1186/s13054-015-0850-8 25887923 PMC4386097

[17] Ostermann M Lumlertgul N. Acute kidney injury in ECMO patients. Crit Care. (2021) 25:313. 10.1186/s13054-021-03676-5 34461966 PMC8405346

[18] Konstantinov IE Saxena P Koniuszko MD Alvarez J Newman MA. Acute massive pulmonary embolism with cardiopulmonary resuscitation: management and results. Tex Heart Inst J. (2007) 34:41–5. Discussion 45–6. 17420792 PMC1847913

[19] Wood KE. Major pulmonary embolism: review of a pathophysiologic approach to the golden hour of hemodynamically significant pulmonary embolism. Chest. (2002) 121:877–905. 10.1378/chest.121.3.877 11888976

[20] Wood KE. Major pulmonary embolism. Crit Care Clin. (2011) 27:885–906. 10.1016/j.ccc.2011.09.002 22082519

[21] Hori D Tanaka M Kohinata T Kimura C Naito K Yamaguchi A. Successful usage of extracorporeal membrane oxygenation as a bridge therapy for acute pulmonary embolism between hospitals. Gen Thorac Cardiovasc Surg. (2010) 58:283–6. 10.1007/s11748-009-0540-z 20549458

[22] Shemin RJ. Surgical embolectomy for massive and submassive pulmonary embolism and pulmonary thromboendarterectomy for chronic thromboembolic pulmonary hypertension. Tech Vasc Interv Radiol. (2017) 20:175–8. 10.1053/j.tvir.2017.07.006 29029711

[23] Chuang CJ Hsu CS. Successful application of extracorporeal membrane oxygenation and pulmonary thromboembolectomy in a patient with a life-threatening pulmonary embolism. Taiwan J Obstet Gynecol. (2015) 54:467–8. 10.1016/j.tjog.2015.06.005 26384077

[24] Aymard T Kadner A Widmer A Basciani R Tevaearai H Weber A. Massive pulmonary embolism: surgical embolectomy versus thrombolytic therapy–should surgical indications be revisited? Eur J Cardiothorac Surg. (2013) 43:90–4. Discussion 94. 10.1093/ejcts/ezs123 22466693

[25] He C Von Segesser LK Kappetein PA Mestres CA Smith JA Choong CK. Acute pulmonary embolectomy. Eur J Cardiothorac Surg. (2013) 43:1087–95. 10.1093/ejcts/ezs605 23220935

[26] Stein PD Alnas M Beemath A Patel NR. Outcome of pulmonary embolectomy. Am J Cardiol. (2007) 99:421–3. 10.1016/j.amjcard.2006.08.050 17261411

[27] Corsi F Lebreton G Brechot N Hekimian G Nieszkowska A Trouillet JL. Life-threatening massive pulmonary embolism rescued by venoarterial-extracorporeal membrane oxygenation. Crit Care. (2017) 21:76. 10.1186/s13054-017-1655-8 28347320 PMC5369216

[28] Pavlovic G Banfi C Tassaux D Peter RE Licker MJ Bendjelid K Giraud R. Peri-operative massive pulmonary embolism management: is veno-arterial ECMO a therapeutic option? Acta Anaesthesiol Scand. (2014) 58:1280–6. 10.1111/aas.12411 25251898

[29] Zeni PT Jr Blank BG Peeler DW. Use of rheolytic thrombectomy in treatment of acute massive pulmonary embolism. J Vasc Interv Radiol. (2003) 14:1511–5. 10.1097/01.RVI.0000099526.29957.EF 14654484

[30] Villalba L Nguyen T Feitosa RL Jr Gunanayagam P Anning N Dwight K. Single-session catheter-directed lysis using adjunctive power-pulse spray with AngioJet for the treatment of acute massive and submassive pulmonary embolism. J Vasc Surg. (2019) 70:1920–26. 10.1016/j.jvs.2019.03.038 31147112

[31] Fontaine AB Borsa JJ Hoffer EK Bloch RD So CR Newton M. Type III heart block with peripheral use of the Angiojet thrombectomy system. J Vasc Interv Radiol. (2001) 12:1223–5. 10.1016/S1051-0443(07)61684-4 11585891

[32] Pelliccia F De Luca A Pasceri V Tanzilli G Speciale G Gaudio C. Safety and outcome of rheolytic thrombectomy for the treatment of acute massive pulmonary embolism. J Invasive Cardiol. (2020) 32:412–6. 33130592 10.25270/jic/20.00173

[33] Vesely TM Williams D Weiss M Hicks M Stainken B Matalon T. Comparison of the angiojet rheolytic catheter to surgical thrombectomy for the treatment of thrombosed hemodialysis grafts. Peripheral AngioJet Clinical Trial. J Vasc Interv Radiol. (1999) 10:1195–205. 10.1016/S1051-0443(99)70220-4 10527197

[34] Kuo WT Gould MK Louie JD Rosenberg JK Sze DY Hofmann LV. Catheter-directed therapy for the treatment of massive pulmonary embolism: systematic review and meta-analysis of modern techniques. J Vasc Interv Radiol. (2009) 20:1431–40. 10.1016/j.jvir.2009.08.002 19875060

[35] Dukkipati R Yang EH Adler S Vintch J. Acute kidney injury caused by intravascular hemolysis after mechanical thrombectomy. Nat Clin Pract Nephrol. (2009) 5:112–6. 10.1038/ncpneph1019 19092794

[36] Wan S Quinlan DJ Agnelli G Eikelboom JW. Thrombolysis compared with heparin for the initial treatment of pulmonary embolism: a meta-analysis of the randomized controlled trials. Circulation. (2004) 110:744–9. 10.1161/01.CIR.0000137826.09715.9C 15262836

[37] Spies C Khandelwal A Smith TH Jolly N Kavinsky CJ. Percutaneous mechanical thrombectomy for massive pulmonary embolism using a conservative treatment strategy. J Interv Cardiol. (2008) 21:566–71. 10.1111/j.1540-8183.2008.00405.x 18973510

